# Predictors of Vaccination Intentions and Behaviour during the COVID-19 Pandemic in Italy

**DOI:** 10.3390/bs13110950

**Published:** 2023-11-19

**Authors:** Amanda Nerini, Mirko Duradoni, Camilla Matera, Andrea Guazzini, Monica Paradisi, Adriele Schembri

**Affiliations:** 1 Department of Education, Languages, Intercultures, Literatures and Psychology, University of Florence, 50135 Florence, Italy; mirko.duradoni@unifi.it (M.D.); camilla.matera@unifi.it (C.M.); andrea.guazzini@unifi.it (A.G.); monica.paradisi@unifi.it (M.P.); 2 School of Psychology, University of Florence, 50135 Florence, Italy; adriele.schembri@stud.unifi.it

**Keywords:** vaccination hesitancy, fear, protection motivation theory, COVID-19 vaccination, health behaviour, prediction, psychology

## Abstract

The present research aimed at understanding individuals’ vaccination intentions and protective behaviours against COVID-19 through two different studies. In Study 1 (N = 213, 73% women; mean age = 24.03) the Protection Motivation Theory model was tested considering the fear of COVID-19 as a possible mediator between threat appraisal (in terms of both health and social life) and intentions to get vaccinated when the vaccination was not yet available. Study 2 (N = 1111, 68.9% women; mean age = 38.33) was conducted when the vaccine became available for the entire population. Through this study, by adopting the 5C model of vaccine hesitancy as a theoretical framework, we aimed to understand how people who got vaccinated and the ones who did not differed, considering fear of vaccination, personality and vaccination hesitancy. In Study 1, social limitations, perceived severity and COVID-19 perceived vulnerability were significantly and positively related to fear of COVID-19. Contrary to what we expected, fear of COVID-19 was not a significant predictor of vaccination intention, which was predicted by both response efficacy and self-efficacy. In Study 2, in line with previous studies, vaccine hesitancy was negatively related to vaccination. More specifically, the social-oriented dimension of collective responsibility was the strongest predictor of effective behaviour. Our findings provide insights into the complexity of vaccine acceptance and emphasise the need for targeted interventions to promote vaccination and mitigate the spread of infectious diseases.

## 1. Introduction

On 11 March 2020, in light of the rapid spread of COVID-19 around the world, the World Health Organization [1] deemed the disease to have achieved pandemic status. On 27 December 2020, following European Medicines Agency approval, the COVID-19 vaccination campaign was launched in Europe to ensure that herd immunity was achieved as quickly as possible [2], which allowed for a gradual and steady relaxation of containment measures. However, there was a significant portion of the population that did not want to receive the COVID-19 vaccination, although it was available. The analysis of factors promoting the intention to get vaccinated is still ongoing and will help health professionals plan interventions that can foster behavioural change in order to ensure as many people are vaccinated as possible. Adopting the Protection Motivation Theory (PMT) [3,4] and the 5C model [5] as theoretical frameworks, the present study aimed to identify factors that might motivate individuals to receive such a vaccination.

According to PMT [3,4] the motivation to protect oneself, considered to be the proximate cause of health behaviour, is affected by threat and coping appraisal. Threat appraisal is determined by perceived threat severity, which refers to the beliefs about the negative consequences that may occur as a result of the maladaptive behaviour, and threat vulnerability, which consists of the probability of being susceptible to the negative consequences of the maladaptive behaviour. Coping appraisal is determined by response efficacy, which refers to the personal perceptions that adaptive behaviour can eliminate or reduce the negative consequences of maladaptive behaviour, and self-efficacy, which refers to the individual’s ability to implement the recommended behaviour. Fear, which is elicited in response to a situation judged as dangerous and towards which a protective action is taken [3], is considered to mediate the relationship between threat appraisal and the intention to implement a protective behaviour [6,7]. In recent years, several studies applied PMT to the intention to be vaccinated against COVID-19, partially confirming its effectiveness in predicting behavioural intention [8,9,10,11,12]. Indeed, among the PMT variables, the response efficacy was found to be significantly related to received vaccination against COVID-19 [8,9,10,12] and seemed to be the most decisive predictor; in contrast, the role of self-efficacy, perceived threat severity, and threat vulnerability is unclear, as these variables have not always emerged as significant predictors of the intention to be vaccinated against COVID-19 [8,9,10,11,12]. Xiao and colleagues [12] found a significant role of the variables related to the coping appraisal in the prediction of the intention to get vaccinated against COVID-19; specifically, response efficacy was the most powerful predictor of the criterion variable, while no significant association emerged between threat appraisal (threat severity and threat vulnerability) and the intention to be vaccinated against COVID-19. In the study by Wang and colleagues [11] on a sample of 3.145 university students, perceived severity of COVID-19 was positively associated with the motivation to get vaccinated; nevertheless, the coping appraisal dimensions of PMT and threat vulnerability were not significant predictors of motivation. Nerini and colleagues [13] showed that the fear of COVID-19 was relevant in motivating people to follow the measures set by the Italian government to reduce the spread of the pandemic and mediated the relationship between threat assessment and intentions. Feelings of fear arising from the perception that COVID-19 was a serious disease that could infect everyone motivated individuals to adopt protective behaviours [13].

Scholars considered fear of vaccination as an important factor in vaccine hesitancy dynamics worldwide [14]. In the first few months of 2021, due to the widespread of fake news (e.g., claims that COVID-19 was harmless and the vaccines were poorly tested and experimental, with hidden side effects) [15,16], fear extended beyond the virus itself to include concerns about the vaccine [17]. The fear of experiencing adverse reactions or developing vaccine-related diseases, together with distrust of pharmaceutical companies and politicians, were common reasons for vaccine refusal [18,19,20,21]. All these factors contributed to the development of vaccine hesitancy, which refers to a hesitation or delay in accepting vaccination, even when vaccination services are readily available [22]. The World Health Organization identified vaccine hesitancy as one of the top 10 threats to global health in 2019 [23]. Apart from fear of vaccination, vaccine hesitancy is associated with socio-demographic (i.e., age and sex) and psychological (e.g., personality) variables [24,25]. Women and younger individuals seemed to be more hesitant and resistant [25]. Moreover, conscientiousness, narcissism, and psychopathy emerged as significant predictors of hesitation in accepting the COVID-19 vaccine [24].

Previous studies about COVID-19 considered a perceived threat and associated feelings of fear as exclusively related to health (e.g., risk of death or long-term consequences). Nevertheless, both fear and vaccination hesitancy might be also influenced by the negative consequences of the pandemic that people perceive with respect not only to their health status, but also to their social life (e.g., limitations to individuals’ social activities). The role of social factors in determining the intention to get vaccinated was considered in Betsch and colleagues’ 5C model [5]. The authors identified five psychological factors as the main antecedents of vaccination behaviour: confidence, complacency, calculation, constraints and collective responsibility. Confidence refers to individuals’ trust in the effectiveness and safety of vaccines, as well as their faith in the recommendations made by health professionals and policymakers. Complacency reflects a low perceived risk and a diminished sense of threat regarding vaccine-preventable diseases. Calculation captures extensive information-seeking behaviour, which can lead to vaccine hesitancy due to exposure to anti-vaccination messages. Constraints encompass both physical and psychological barriers to vaccine accessibility (e.g., geographical, financial, and comprehension issues). Collective responsibility refers to the willingness to protect others through vaccination, contributing to herd immunity. Unlike the other four factors, collective responsibility has a strong social valence. Individuals with high levels of confidence and collective responsibility tend to exhibit positive attitudes towards vaccination; in contrast, those with high levels of complacency and constraints are more likely to hold negative attitudes. Empirical evidence regarding the calculation factor remains inconclusive [5].

## 2. Research Overview

Even though previous studies confirmed the validity of PMT in predicting the intention to engage in preventive health behaviour, mixed results have emerged about the role played by PMT dimensions in the prediction of the intention to get vaccinated against COVID-19. In addition, in these studies, threat appraisal was considered exclusively with regard to the negative consequences of being infected with COVID-19 (such as risk of death or long-term consequences). To the best of our knowledge, no study assessed the perception of threat with regard to the negative consequences that may occur in terms of limitations to one’s social life. As COVID-19 pandemic was an unprecedented event that has affected the entire globe, politicians, health experts, but also pundits and public opinionists filled the media with different, and sometimes opposing, positions about containment measures and vaccination. In order to motivate people to protect themselves and others, it is crucial to understand if and how classic theoretical models are able to explain individuals’ vaccination intentions and behaviours and if factors that are more related to the broader social context should be considered together with more strictly cognitive ones.

Starting from these considerations, we conducted two studies: a preliminary study (study 1) aimed at analysing the intention to get vaccinated in a specific segment of the population (young adults) for whom the vaccine, at the time in which the study was conducted, was not yet available; a second study aimed to understand individuals’ vaccination behaviour when the vaccine became available for the entire population, so that we moved from analysing the intention to get vaccinated to analysing people who had actually received the vaccination.

Specifically, in the first study, the PMT model was used to explain the intention to get vaccinated when the vaccination was not yet available. We also analysed the mediational role of fear of COVID-19 in the relationship between threat appraisal and intentions, considering threats related not only to one’s health, but also to one’s social life.

In the second study, we examined vaccination hesitancy in people who got and did not get vaccinated. By adopting the 5C model of vaccine hesitancy as a theoretical framework, we considered factors that, although still cognitive in nature, capture the influence of the social context on the decision to receive the COVID-19 vaccine (e.g., collective responsibility). Moving beyond Study 1’s emphasis on fear of the disease, Study 2 focused on the fear associated with the recommended response, namely vaccination. Specifically, we examined the role of vaccination fear in relation to vaccine hesitancy dimensions, along with sociodemographic variables such as sex and age, as well as personality traits.

In Study 1, we tested the following hypotheses. Both COVID-19 threat appraisal (i.e., COVID-19 perceived severity and COVID-19 perceived vulnerability) and social limitations threat appraisal (i.e., social limitations perceived severity and social limitations perceived vulnerability) would predict the intention to get vaccinated via fear of COVID-19 (Hypothesis 1). Coping appraisal (i.e., response efficacy and self-efficacy) would be directly associated with the intention to get vaccinated (Hypothesis 2).

In Study 2, we tested the following hypotheses. Vaccine hesitancy would be negatively associated with actual vaccination (Hypotheses 3). Vaccine fear would be positively associated with vaccine hesitancy, even after controlling for participants’ age, sex, and personality traits based on the 5C model (Hypotheses 4).

## 3. Study 1

### 3.1. Methods

#### 3.1.1. Participants and Procedure

The participants were 213 Italian young adults (73% women; mean age = 24.03, *SD* = 3.03). As we planned to analyse the data through path analysis, following the indications by Weston and Gore [26], we aimed to recruit a sample of more than 200 participants. To be eligible for the study, the participants had to be aged between 18 and 30 years, as vaccination against COVID-19 was not available for this group in Italy at the time the questionnaire was administered (spring of 2021). Despite that, as some groups of professionals had been vaccinated independent of age (e.g., teachers, health professionals), to be eligible for the study, participants had to confirm that they had not received any doses of vaccination against COVID-19.

An online anonymous survey was shared through social networking sites (i.e., Facebook groups, Instagram, LinkedIn) between 21 May 2021 and 14 June 2021, inviting participants to take part in a study on Italian youth intentions of getting vaccinated against COVID-19. No incentive was provided, and all participants gave their informed consent to the study procedures, which adhered to the Helsinki Declaration, Italian legal requirements on privacy and informed consent (Law Decree DL-101/2018), EU regulations (2016/699) and guidelines from the American Psychological Association (APA). The Ethical Committee of the University of Florence approved the study procedures (Ref. No. 0096243). The survey took approximately 15 min to complete. To ensure participants had complete control over whether to submit their responses or not, we did not record responses from participants who did not complete the survey. 

#### 3.1.2. Measures

For the following measures, all the items were rated on a five-point Likert scale (1 = strongly disagree; 5 = strongly agree). Higher scores indicated higher levels in the measured variables.

COVID-19 and social limitations threat appraisals. COVID-19 and social limitations threat appraisals were assessed in terms of both perceived severity and vulnerability through ten items adapted from previous studies conducted in different countries, including Italy [13,27,28]. COVID-19 perceived severity was measured using a three-item scale (e.g., “Those who do not get vaccinated in the event of contagion can put the health of their loved ones at risk”; alpha = 0.86) and COVID-19 perceived vulnerability through two items (e.g., “The possibility that I could contract COVID-19 is high”; *r* = 0.57; *p* < 0.001). Social limitations perceived severity was measured using a three-item scale (e.g., “those who do not get vaccinated have a more limited social life”; alpha = 0.84) and social limitations perceived vulnerability through two items (e.g., “I suffer as I feel my social life is limited”; *r* = 0.45; *p* < 0.001).

Coping Appraisal. Coping appraisal was measured through eight items adapted from previous studies [13,27,28] intended to capture both response efficacy and self-efficacy. Response efficacy was assessed using a four-item scale (e.g., “Vaccination will reduce the likelihood of getting sick”; alpha = 0.88), as well as self-efficacy (e.g., “When vaccination will be available, I will be able to get vaccinated”; alpha = 0.86).

Fear of COVID-19. Feelings of fear were assessed using the 7-item (e.g., “My heart races when I think I might get COVID-19”; alpha = 0.86) Italian version [29] of the Fear of COVID-19 Scale (FCV-19S) [30].

Intentions to get vaccinated. The intention to get vaccinated against COVID-19 was measured through three items (e.g., “When it will be possible, I will make myself available to get vaccinated against COVID-19”; alpha = 0.93) adapted from previous studies conducted in different countries, including Italy [13,27,28].

Sociodemographic details. All the participants reported their age, sex and nationality.

#### 3.1.3. Data Analyses

We tested a model in which both COVID-19 and social limitations threat appraisals were posited as predictors of the intention to get vaccinated via fear of COVID-19, while coping appraisal was directly associated with the intention to perform the behaviour. We used bootstrapping to test mediation by estimating the presence and size of the indirect (i.e., mediated) effects [31]. As specified in the paragraph devoted to participants and procedure, we considered that a sample size of at least 200 participants would be required [26]. We adopted the following goodness-of-fit indices: the *χ*^2^/df ratio, a good score of which was two or below; the Comparative Fit Index (CFI); the Incremental Fit Index (IFI), the value of which should be higher than 0.95; the Normed Fit Index (NFI), a good score of which is more than 0.90; the Root Mean Square Error of Approximation (RMSEA); a 90% confidence interval for RMSEA (RMSEA 90% CI); and the Standardised Root Mean square Residual (SRMR). RMSEA and SRMR are considered acceptable if they are 0.08 or lower [32].

### 3.2. Results

Descriptive statistics are displayed in Table 1. For almost all variables’ participants reported a medium–high mean score.

Table 2 presents the intercorrelations between variables, which were all positive and significant. The strongest intercorrelations were found between the two coping appraisals variables (self-efficacy and response efficacy) and the intention to get vaccinated. Fear was strongly associated with COVID-19 perceived vulnerability.

**Figure 1:**
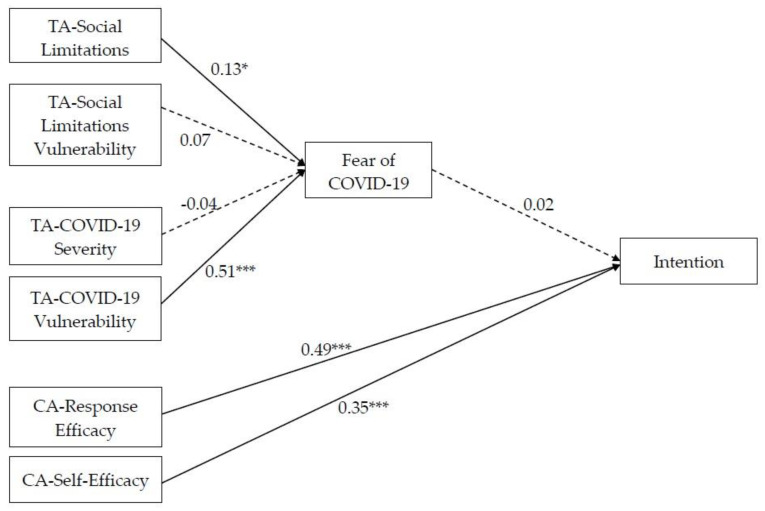
Final model. Note: * *p* < 0.05; *** *p* < 0.001. TA = threat appraisal; CA = coping appraisal.

The proposed model (Figure 1) fit very well with the data [*χ*^2^/df = 1.98, *p* = 0.06; RMSEA = 0.07 (CI = 0.01; 0.12); SRMR = 0.02; CFI = 0.99; IFI = 0.99; NFI = 0.98] and accounted for an important percentage of the variance of intentions (59%).

Partially in line with Hypothesis 1, people who believed that COVID-19 could have negative consequences for their social life and perceived themselves susceptible to the negative consequences of contracting the virus were more afraid of contracting COVID-19. Specifically, social limitations, perceived severity and COVID-19 perceived vulnerability were significantly and positively related to fear of COVID-19, and COVID-19 perceived vulnerability emerged as the strongest predictor of fear. Contrary to Hypothesis 1, COVID-19 perceived severity and social limitations perceived vulnerability were not significantly related to fear of COVID-19. Moreover, fear did not predict the intention to get vaccinated.

In line with Hypothesis 2, individuals who were confident regarding their ability to get vaccinated and perceived that vaccine could reduce the negative consequences of COVID-19 were more predisposed to implement this adaptive behaviour. Indeed, both perceived self-efficacy and response efficacy were significantly and positively associated with the intention to get vaccinated.

## 4. Study 2

### 4.1. Methods

#### 4.1.1. Participants and Procedure

The data were collected between 20 December 2021 and 10 January 2022, after the vaccine became available but before it became mandatory. The final sample was composed of 1111 Italian-speaking participants who completed an online survey (68.9% women; mean age = 38.33, *SD* = 13.94). For some analyses (i.e., discriminant analysis and hierarchical regression), we had to exclude approximately 5% of the initial sample due to the necessity of maintaining a balanced representation across different sex groups in the analyses, particularly when considering individuals who identified as transgender or non-binary. Given the limitations in obtaining a sufficiently large sample for these specific sex identities, we opted to exclude these cases from the analyses while retaining a focus on age and binary sex. This was to prevent the introduction of substantial biases due to the inherent imbalance in the group sizes. Moreover, since we aimed to detect even relatively small effects while modelling for the impact of sociodemographic and personality variables, we conducted a preliminary power analysis. The analysis revealed that, given the chosen set of predictors, a sample size of 1000 participants would be necessary to achieve a statistical power of 0.90, enabling us to identify even relatively small effects (f^2^ = 0.02) as statistically significant. Since we were able to secure more than 1000 participants, we deemed our sample size adequate. The procedure was the same as in Study 1. The survey took approximately 20 min to complete. To ensure participants had complete control over whether to submit their responses or not, we did not record responses from participants who did not complete the survey. The Ethical Committee of the University of Florence approved the study procedures (Ref. No. 0092811).

#### 4.1.2. Measures

In the following measures, all the items were rated on a five-point Likert scale (1 = strongly disagree; 5 = strongly agree). Higher scores indicated higher levels in the measured variables.

Personality. The Italian version [33] of the Ten-Item Personality Inventory (I-TIPI) [34] was used. The scale is composed of ten items assessing five dimensions: extraversion (e.g., extraverted, enthusiastic), agreeableness (e.g., sympathetic, warm), conscientiousness (e.g., dependable, self-disciplined), emotional stability (e.g., calm, emotionally stable) and openness to experience (e.g., open to new experiences, complex).

Fear of vaccination. The Italian version [17] of the Fear of Vaccination Scale (VFS-6) [35] was used. The scale consists of six items comprising two dimensions: cognitive (alpha = 0.70) and somatic (alpha = 0.74).

Vaccination hesitancy. The 5-C scale [5] was used to assess vaccine hesitancy. The scale consists of 15 items which comprise five dimensions: confidence (i.e., trust in the effectiveness and safety of vaccines); complacency (i.e., vaccination not deemed as a necessary preventive action); constraints (i.e., reporting issues that prevent one from getting vaccinated); calculation (i.e., extensive engagement in evaluating the risks of infections and vaccination to derive a good decision); and collective responsibility (i.e., the willingness to protect others through one’s own vaccination). Cronbach’s α for the 5-C scale ranges from 0.67 to 0.88.

Sociodemographic details. All the participants reported their age, sex and nationality.

### 4.2. Results

Descriptive statistics disaggregated by sex are presented in Table 3.

To test how much vaccine hesitancy, in its five components, was related to actual vaccination, we relied on discriminant analysis [36]. This technique allowed us to investigate the efficacy of a linear combination of vaccine hesitancy dimensions in differentiating between vaccinated and unvaccinated people. The data were therefore analysed using stepwise discriminant analysis, excluding non-statistically significant factors. The five selected indicators were entered concurrently in the discriminant function analysis. The linear equation performed quite well after excluding the vaccination hesitancy constraints dimension which resulted as a non-statistically significant factor. In fact, Wilk’s Lambda (Λ = 0.58), chi-square values (*χ*^2^_(4)_ = 577.8), and the level of statistical significance (*p* < 0.001) provided robust support for a linear discriminating function, together with a canonical correlation of 0.65 and a predictive accuracy of the model of 88.1%. The relative contribution to the equation of each variable is reported in Table 4.

The derived function was mainly composed of collective responsibility (β = 0.57) and confidence dimensions (β = 0.31). The complacency dimension appeared to make a secondary contribution to the equation (β = −0.23) while calculation was of less importance (β = −0.12).

Subsequently, we performed multiple regression to assess the best set of predictors for each of the vaccine hesitancy dimensions that emerged connected to actual vaccination in discriminant analysis (see Table 5).

As shown in Table 5, the role of both socio-demographic variables (i.e., age and sex) and personality traits was quite limited in terms of the number of statistically significant results and their effect size. Age was positively correlated with the extent of calculation, suggesting that older participants tended to engage in a more thorough evaluation of the advantages and disadvantages of vaccination. Conversely, age exhibited a negative correlation with collective responsibility, indicating that older individuals might be somewhat less inclined to protect others through their own vaccination efforts.

Regarding gender, cisgender women displayed lower levels of hesitancy in two out of four dimensions of vaccination hesitancy. In contrast, traits such as extraversion and conscientiousness did not demonstrate a significant association with vaccination hesitancy, particularly when accounting for vaccine fear. Similarly, openness showed no consistent relationship with vaccination hesitancy, except for a positive association with a higher level of complacency.

Conversely, agreeableness was linked to reduced complacency and increased calculation. Lastly, neuroticism consistently exhibited a negative association with vaccination hesitancy, implying that individuals with higher neuroticism scores tend to have lower levels of vaccination hesitancy. Instead, vaccination fear, mostly in its cognitive component, appeared to substantially explain the variance of the vaccination hesitancy subscales (more than 50% for confidence, complacency and collective responsibility; 17% for calculation).

## 5. Discussion

Our first study showed that vaccination intentions were significantly predicted by coping appraisal determinants, namely response efficacy and self-efficacy, in line with PMT [3]. These findings suggest that people who believed the vaccine was useful and felt more confident regarding their ability to get vaccinated were also more willing to perform the behaviour. Notably, our findings showed that fear was associated not only with the perception of negative health consequences of COVID-19, but also for one’s social life. Individuals who were worried about the consequences of not getting vaccinated in terms of limitations to mobility and social experiences reported higher levels of fear. Nevertheless, contrary to our hypothesis, fear of COVID-19 did not predict vaccination intention. While in previous research, fear of COVID-19 mediated the link between threat appraisal and intention to follow the measures to reduce the spread of the virus [13], this same role did not emerge in relation to vaccination intention. This unexpected result may be attributed to the age of the participants, for whom levels of fear might be not so high to trigger motivation. Moreover, during the period in which the data were collected, the situation might have been evaluated as less dangerous than one year before, when Nerini and colleagues conducted their study (2021).

In Study 2, we moved from analysing the intention to get vaccinated to actual vaccination. In line with previous studies, vaccine hesitancy was negatively related to vaccination [37,38]. More specifically, the more social-oriented dimension of collective responsibility was the strongest predictor of vaccination, highlighting that vaccination should not be considered a pure individual cognitive-based phenomenon. Indeed, the individual and social sphere are intertwined and interdependent with one another with regard to defining human behaviour [39]. Notably, the cognitive component of vaccine fear was strongly and negatively associated with the collective responsibility dimension of vaccine hesitancy, so that perceived social pressure to comply with vaccination (e.g., being a good and respected member of the community) might push people to overlook their fears [40,41].

We should acknowledge some limitations of our studies. First, in Study 1, the perceived threat might have changed during the data collection period. Future studies could explore the temporal dynamics of fear in relation to vaccination intention to better understand the role of fear as a mediator. Second, although we considered the role of some social factors, we focused mostly on individual-level psychological ones. Future studies could investigate the interplay between individual and sociocultural variables (e.g., social norms, peer influence, community support), together with structural ones, in shaping vaccination behaviour. As was the case for other non-pharmaceutical interventions (NPI) implemented during the pandemic, such as contact tracing methods, the influence of the social context emerged as a pivotal factor in either encouraging or impeding the adoption of specific behaviors. This context likely also contributed to the normalisation of certain fears and beliefs [42]. Moreover, the correlational nature of the studies limits causal interpretations. Longitudinal or experimental designs could be employed to establish causal and temporal relationships among variables. Finally, the present studies focused on specific populations and contexts. Replication of the findings would contribute to their generalisability, thus providing a more comprehensive understanding of vaccination behaviour.

In conclusion, our research contributed to the identification of predictors of vaccination behaviour during the COVID-19 pandemic. Coping appraisal determinants, but not fear, emerged as predictors of vaccination intention when the vaccine was not available. When vaccination was possible, we observed a negative relationship between vaccine hesitancy and actual vaccination, with collective responsibility being a key predictor. These findings provide insights into the complexity of vaccine acceptance and emphasise the need for targeted interventions which consider not only individual but also social factors to promote vaccination and mitigate the spread of infectious diseases. A focus on social and interpersonal factors can be useful not only for increasing vaccination levels, but also for improving people’s wellbeing during periods in which social life is limited [43]. More specifically, the results suggested that messages emphasising the aspect of collective responsibility towards vaccination might be highly desirable. Furthermore, by incorporating references to vaccine safety (confidence) and crafting messages and interventions that reassure individuals about the exaggeration of the likelihood and severity of adverse outcomes resulting from vaccination, along with providing cognitive coping tools to address such fears, we should be able to achieve more effective and evidence-based health communication. Finally, effective messages should address beliefs related to one’s ability in performing the recommended behavior (i.e., get vaccinated), describing it as an easy behavior to implement, thus making people confident that they can get vaccinated without too much effort and difficulty.

## Figures and Tables

**Table 1:** Descriptive statistics: Observed scores (*Min* and *Max*), means (*M*) and standard deviations (*SD*) (N = 213).

Variables	*Min*–*Max*	*M* (*SD*)
TA—Social limitations Severity	1–5	3.94 (1.10)
TA—Social limitations Vulnerability	1–5	4.38 (0.69)
TA—COVID-19 Severity	1–5	4.59 (0.72)
TA—COVID-19 Vulnerability	1–5	3.14 (1.03)
CA—Self-efficacy	1–5	4.33 (0.81)
CA—Response Efficacy	1–5	4.29 (0.87)
Fear of COVID-19	1–5	2.47 (0.86)
Intention to get vaccinated	1–5	4.54 (0.89)

Note: TA = threat appraisal; CA = coping appraisal.

**Table 2:** Intercorrelation between all variables (N = 213).

Variables	1.	2.	3.	4.	5.	6.	7.
1. TA—Social limitations Vulnerability	-						
2. TA—Social limitations Severity	0.23 ***	-					
3. TA—COVID-19 Severity	0.23 ***	0.50 ***	-				
4. TA—COVID-19 Vulnerability	0.23 ***	0.33 ***	0.32 ***	-			
5. CA—Self-efficacy	0.29 ***	0.42 ***	0.42 ***	0.29 ***	-		
6. CA—Response Efficacy	0.36 ***	0.60 ***	0.57 ***	0.37 ***	0.62 ***	-	
7. Fear of COVID-19	0.22 ***	0.27 ***	0.20 **	0.59 ***	0.19 **	0.35 ***	-
8. Intention to get vaccinated	0.24 ***	0.47 ***	0.47 ***	0.21 **	0.66 ***	0.71 ***	0.26 ***

Note: ** *p* < 0.01; *** *p* < 0.001. TA = threat appraisal; CA = coping appraisal.

**Table 3:** Descriptive statistics of the collected variables for the entire sample and disaggregated by sex.

Variables	Entire Sample	Females	Males
	*M* (*SD*)	*M* (*SD*)	*M* (*SD*)
Age	38.33 (13.94)	38.05 (13.82)	38.67 (14.44)
Extraversion	8.19 (2.76)	8.38 (2.85)	7.74 (2.50)
Agreeableness	10.38 (2.03)	10.49 (2.01)	10.05 (2.08)
Conscientiousness	10.42 (2.36)	10.57 (2.28)	10.12 (2.44)
Neuroticism	7.62 (2.77)	7.90 (2.73)	6.94 (2.74)
Openness	9.07 (2.12)	9.09 (2.09)	4.37 (2.31)
VF—Cognitive	7.61 (4.07)	7.74 (4.19)	6.94 (3.66)
VF—Somatic	5.00 (3.01)	5.18 (3.19)	4.37 (2.31)
VH—Confidence	10.21 (1.35)	10.18 (3.30)	10.53 (3.46)
VH—Complacency	5.44 (2.83)	5.17 (2.60)	5.94 (3.20)
VH—Constraints	4.77 (2.29)	4.66 (2.20)	4.82 (2.35)
VH—Calculation	11.03 (2.65)	11.00 (2.53)	11.00 (2.91)
VH—Collective responsibility	11.95 (3.00)	12.15 (2.84)	11.66 (3.26)

Note: *M* = Mean; *SD* = standard deviation; VF = vaccination fear; VH = vaccination hesitancy.

**Table 4:** Standardised discriminant function coefficients.

Predictors	Coefficient
VH Confidence	0.31
VH Complacency	−0.23
VH Constraints	ns
VH Calculation	−0.12
VH Collective responsibility	0.57

Note. N = 1061; VH = vaccination hesitancy; ns = not statistically significant.

**Table 5:** Multiple regression of identified predictors with vaccine hesitancy dimensions.

Predictors	VH Confidence	VH Complacency	VH Calculation	VH Collective Responsibility
Age	−0.04	−0.01	0.07 *	−0.06 *
Sex	0.01	−0.16 ***	−0.04	0.12 ***
Extraversion	0.02	0.01	0.02	−0.01
Agreeableness	0.01	−0.06 *	0.07 *	0.02
Conscientiousness	0.02	−0.02	0.04	0.01
Neuroticism	0.06 **	−0.16 **	−0.02	0.12 ***
Openness	−0.02	0.05 *	0.01	−0.02
VF—Cognitive	−0.76 ***	0.57 ***	0.38 ***	−0.60 ***
VF—Somatic	−0.02	0.11 ***	0.01	−0.11 ***
R^2^	0.60	0.46	0.17	0.49

Note: N = 1061; * *p* < 0.05; ** *p* < 0.01; *** *p* < 0.001; VH = vaccination hesitancy; VF = vaccination fear scale.

## Data Availability

The data that support the findings of this study are available from the corresponding author upon reasonable request.
